# The Therapeutics of “Horizontal Position”

**Published:** 1885-04

**Authors:** 


### THE THERAPEUTICS OF "HORIZONTAL POSITION."

Dr. R. H. Gunning, of Edinburg, tells us that it is enough to look at the veins on the back of the hand or inside of the leg, to see the effects of hydrostatic pressure. The limbs being perpendicular, the veins swell; placed horizontally, they become again normal. If so in the limbs where the veins have valves, more so in the veins where there are no valves, as in the lower intestine and in the reproductive parts. How easy to prevent varix, varicocele, piles, and leucorrhea, by reclining sufficiently; or to develop them by overstanding or over-walking. This is what he thinks is not sufficiently estimated in books nor in practice. Too much is expected from local applications or operations of one kind or another, and too little is trusted to the help of position, or physical law.

Then we must not forget that the force of the heart and general circulation is also diminished by the recumbent position. The pulse increases in frequency by sitting up, and more by standing up.—*Med. and Surg. Reporter.*

